# Anti-Reflectance Optimization of Secondary Nanostructured Black Silicon Grown on Micro-Structured Arrays

**DOI:** 10.3390/mi9080385

**Published:** 2018-08-02

**Authors:** Xiao Tan, Zhi Tao, Mingxing Yu, Hanxiao Wu, Haiwang Li

**Affiliations:** 1 School of Energy and Power Engineering, Beihang University, Beijing 100191, China; by1404143@buaa.edu.cn (X.T.); tao_zhi@buaa.edu.cn (Z.T.); yumingxing1314@126.com (M.Y.); hanxiao_wu_buaa@163.com (H.W.); 2 National Key Laboratory of Science and Technology on Aero Engine Aero-Thermodynamics, Beijing 100191, China; 3 The Collaborative Innovation Center for Advanced Aero-Engines of China, Beijing 100191, China

**Keywords:** absorption, black silicon, secondary nanostructures

## Abstract

Owing to its extremely low light absorption, black silicon has been widely investigated and reported in recent years, and simultaneously applied to various disciplines. Black silicon is, in general, fabricated on flat surfaces based on the silicon substrate. However, with three normal fabrication methods—plasma dry etching, metal-assisted wet etching, and femtosecond laser pulse etching—black silicon cannot perform easily due to its lowest absorption and thus some studies remained in the laboratory stage. This paper puts forward a novel secondary nanostructured black silicon, which uses the dry-wet hybrid fabrication method to achieve secondary nanostructures. In consideration of the influence of the structure’s size, this paper fabricated different sizes of secondary nanostructured black silicon and compared their absorptions with each other. A total of 0.5% reflectance and 98% absorption efficiency of the pit sample were achieved with a diameter of 117.1 μm and a depth of 72.6 μm. In addition, the variation tendency of the absorption efficiency is not solely monotone increasing or monotone decreasing, but firstly increasing and then decreasing. By using a statistical image processing method, nanostructures with diameters between 20 and 30 nm are the majority and nanostructures with a diameter between 10 and 40 nm account for 81% of the diameters.

## 1. Introduction

High reflectance, or so-called low absorption, badly limits the applications of silicon-based photon sensitive and optical devices. Since 1995, in order to reduce the reflectance of silicon surfaces, black silicon was studied with SF_6_/O_2_ plasma and was proposed as a tool to identify the optimal conditions for vertical silicon deep etching [1]. Since then, three methods for black silicon fabrication were developed, which were plasma dry etching, metal-assisted wet etching, and femtosecond laser pulse etching.

As for plasma dry etching, Zaidi et al. studied a solar cell textured by reactive ion etching (RIE), but paid little attention to the secondary nanostructures [2]. The plasma dry etching method is now popular in increasing the bactericidal efficiency due to high density nanostructures [3,4]. Temperature is an important parameter that influences the growth conditions of nanostructures in dry etching. While black silicon can generally be fabricated at low (−40 to −30 °C) [5] or even cryogenic temperatures [6], Pezoldt et al. presented nanostructure fabrication at temperatures between 20 and 30 °C [7]. However, fabrication at temperatures between −30 and 20 °C, i.e., the operating temperature for a normal RIE machine or ICP (inductively coupled plasma), is rarely reported.

The metal-assisted wet etching method typically uses Ag or Au for nanoparticle catalysis and HF for etching substances [8,9,10,11,12], paying little attention to secondary nanostructures like plasma dry etching. Apart from the early references, recent papers have also rarely investigated secondary nanostructures [13,14,15,16,17]. Jia et al. considered that a better surface structure would increase the performance of black silicon and thus they smoothed the surface with NaOH after wet etching [18]. As a result, the smoothed surface indicated the lowest reflectance among the samples. In addition to alkaline treatment, acid treatment after wet etching was investigated but showed a higher reflectance than the no acid treatment sample [19]. Those two studies noticed that surface modification could influence the reflectance of black silicon. However, they did not find the importance of secondary nanostructures. The metal-assisted wet etching method is a simple and quick approach for producing a large amount of nanostructure grass layers. However, the wet etching method may cause large area defects and is less stable than the dry etching method. Hsu et al. investigated the fabrication and characteristics of black silicon for solar cell applications before 2014 and wrote an overview of them [20]. In the same year, Liu reviewed the properties of solar energy applications using black silicon [21]. Both studies recognized black silicon as being promising for energy conservation and collection. In addition to optical applications [22,23,24], other fields, including heat transfer [25], photoelectrochemical [26], electroosmotic flow in microchannel [27], etc., also began to take full advantage of black silicon.

Lv et al. reviewed the recent achievements and applications of black silicon with femtosecond laser pulses [28]. In this review, black silicon was applied to many fields, such as photodiodes [29,30,31,32], photodetectors [33,34,35,36,37], solar cells [38,39,40,41,42,43,44,45,46,47,48,49], field emission [50,51,52,53], luminescence [54,55], hydrophobic surface [56,57,58], etc. They pointed out that the laser-irradiated process is relatively slow compared to other methods and that the laser-irradiated process needs to be studied further. Liu et al. reported that the micro-ripple and micro-bead structures with femtosecond laser pulses in a nitrogen (N_2_) atmosphere could increase the absorption of N-doped silicon with wavelengths from 1.1 to 2.5 µm [59]. Zhan et al. studied the porous microstructures fabricated in the air and increased the absorption by changing the scan parameters [60]. Liu fabricated non-doped black silicon in an argon (Ar) atmosphere and increased the absorption of it by changing the energy of the laser [61].

Kim et al. reported a secondary structure of black silicon with nanostructures on the bottom of the micro trenches and no grass in the profiles [62]. However, they cared more about the heat transfer characteristics rather than optical properties. Although various methods for nanostructure fabrication have been investigated in detail over the past few decades, rarely have articles paid attention to the secondary structures. Therefore, this paper focuses on the fabrication of micro-nano secondary structures and their reflectance values. In addition, micro-nano secondary structures are beneficial for absorbing the infrared spectrum of solar radiation, from which a solar cell can absorb more thermal energy. Such an absorption may also reduce the forbidden gap of silicon because a negative correlation exists between temperature and the forbidden gap value. In this paper, secondary structures with size optimization showed a better performance (lower reflectance) than single structures using the same etching method [63]. The results of using hybrid structures indicated that the reflectance kept low and stable from 300 nm–1000 nm, but that of using single structures in others may show that the reflectance increased for small wavelengths [64,65,66].

Kanmin et al. developed a unique nano- and microwire hybrid structure by selectively modifying only the tops of microwires using metal-assisted chemical etching [67]. The proposed nano/micro hybrid structure not only minimizes surface recombination but also absorbs 97% of incident light under AM 1.5 G illumination, demonstrating outstanding light absorption compared to that of planar (59%) and microwire arrays (85%). Kwang-tae et al. reported a combined wire structure, made up of longer periodic Si microwires and short nanoneedles, which was prepared to enhance light absorption using one-step plasma etching via lithographical patterning [68]. The combined wire array exhibited light absorption of up to ≈97.6% from 300 to 1100 nm without an anti-reflection coating. The shape of the combined wire arrays was cylindrical with a high ratio of depth and width that was not be fabricated easily. In-Ji et al. investigated how silicon nanowires affected the photovoltaic performance of silicon solar cells with a pyramid textured surface [69]. They obtained a high absorption surface with a 95% efficiency of absorption. This paper investigated secondary structures based on these three papers with further size optimization.

Reflectance for normal incidence was measured on a UV3600 spectrophotometer (Shimadzu, Kyoto, Japan). The morphology and structures of the samples were characterized by a scanning electron microscope (SEM), from SEC (Suwon, Gyeonggi-do, Korea) and the Zeiss Company (Oberkochen, Germany).

## 2. Fabrication

**Figure 1:**
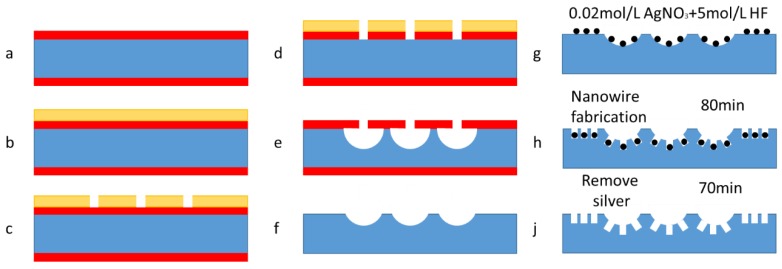
The process flow of secondary structures. (**a**) Piranha washing for 10 min, (**b**) spin coat photoresist, and prebake 30 min in nitrogen atmosphere oven, (**c**) pattern photoresist and post bake for 15 min in a nitrogen atmosphere oven, (**d**) using a buffer oxide etching fluid to etch silicon oxide, (**e**) piranha washing for 10 min to remove the photoresist, (**f**) silicon dioxide removed, (**g**) solution preparation, (**h**) solution (AgNO3/0.02-mol/L: HF/5-mol/L) configuring and soaking for 80 min, and (**j**) solution (HNO3/65%) configuring and soaking for 70 min.

The aim of this study is to fabricate a kind of black silicon with a high light-abortion efficiency. Two kinds of samples were fabricated and their size details were different, as shown in Figure 1 and Table 1 below. These structures were actually secondary structures and they were designed to absorb more light than the wafers with single micro- or nanostructures. In these experiments, 4-inch silicon wafers with a N-doped crystal orientation of 100, a thickness of 500 μm, and silicon oxide with a thickness of 1 μm were used. Fabrication included two steps. The first step used high-energy, high-density plasma for isotropic etching with SF_6_/O_2_ (130 sccm/13 sccm) to manufacture microstructures, tips, and pits. As shown in Figure 1, the first step was to fabricate the microstructure, including the micro tips and pits. The details of the first-class structure’s process flow are as below: (a) piranha (H_2_SO_4_/98%:H_2_O_2_/30% = 3:1) washing for 10 min, (b) spin coat photoresist, S1813, at 3000 rpm and prebake for 30 min in a nitrogen atmosphere oven, (c) pattern photoresist with 10 mJ/cm^2^ ultraviolet light for 3 s and post bake for 15 min in a nitrogen atmosphere oven, (d) using a buffer oxide etching fluid (HF:NH_4_F = 1:5) to etch silicon oxide without the destruction of photoresist, (e) piranha (H_2_SO_4_/98%:H_2_O_2_/30% = 3:1) washing for 10 min to remove the photoresist, and finally (f) after 5 min of supersonic treatment in acetone and ethanol, the silicon wafers with micro tips or pits were fabricated. After first-class-structure fabrication, the samples were fabricated using the metal-assisted catalyst etching method, with the aim of growing secondary nanostructures on the first-class structures. In the second step, the nanostructures were fabricated on the tips and pits, as well as on a flat sample for comparison. Two types of secondary nanostructured samples and two flat samples were fabricated. It was performed to obtain microstructures with tips, pits, and a flat surface. The wet etching method process in the second step was as follows: (g) piranha (H_2_SO_4_/98%:H_2_O_2_/30% = 3:1) washing for 10 min, (h) solution (AgNO_3_/0.02-mol/L: HF/5-mol/L) configuring and soaking for 80 min, and (j) solution (HNO_3_/65%) configuring and soaking for 70 min.

**Figure 2:**
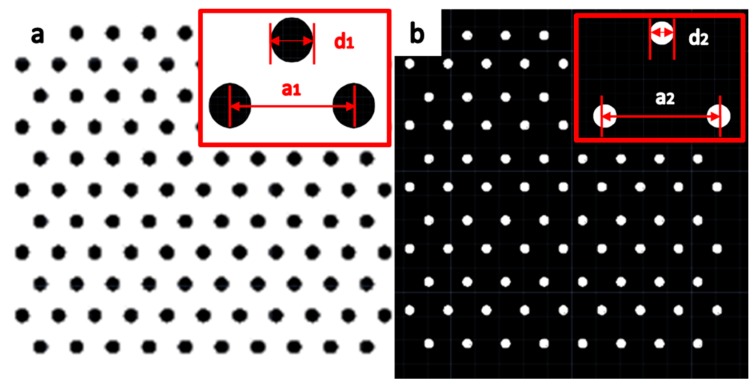
The schematic diagram of the mask size parameter of the two samples. (**a**) Array tips sample mask. (**b**) Array pits sample mask.

Table 1 shows the group number and size details. One kind of sample mask was filled with array tips as shown in Figure 2a, and another was filled with array pits as shown in Figure 2b. In Figure 2a,b, black patterns denote mask layers that can hardly be etched by plasma and the white patterns denote areas with no masks. *d*_1_ and *d*_2_ denote the diameter of the patterns, and *a*_1_ and *a*_2_ denote the distance between the two circles. Every group of three neighboring circles made up an equilateral triangle.

**Figure 3:**
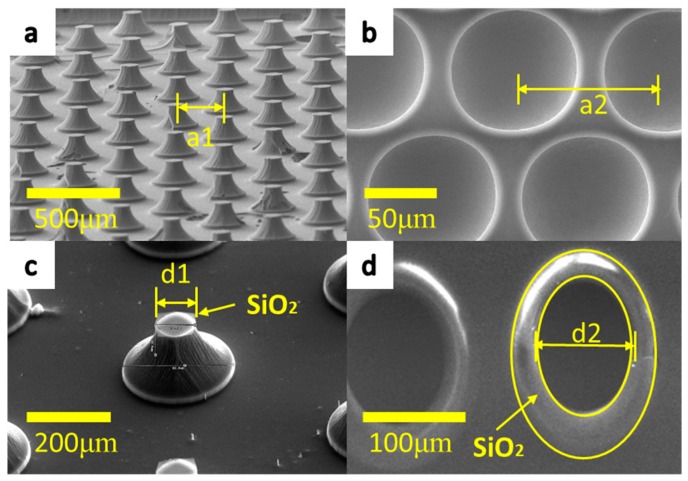
The schematic diagram of parameters *d*_1_, *a*_1_, *d*_2_, and *a*_2_ in the SEM view. (**a**) *a*_1_, the distance between the two center point of tips masks is 240 μm, that is, the No. 5 tip sample, (**b**) *a*_2_, the distance between the two center point of pits masks is 100 μm, that is, the No. 1 pit sample, (**c**) *d*_1_, the distance between the two center point of tips masks, is 33.6 μm, that is, the No. 2 tip sample, (**d**) *d*_2_, the distance between the two center point of pits masks is 100 μm, that is, the No. 2 pit sample.

As shown in the figure below, the micro tips array is indicated in Figure 3a and this picture was filmed by SEM in a low magnification view. The micro pits array is indicated in Figure 3b and this picture was filmed by SEM in a low magnification view. Figure 3c, d indicate that silicon dioxide, as the mask layer, remained in the surface after dry etching. The numbers of *a*_1_, *a*_2_, *d*_1_, and *d*_2_ are shown in Figure 3, coming from the No. 2 and No. 5 samples.

As shown in Figure 1e, the tips or pits were etched under the protection of a silicon dioxide mask. In order to fabricate hemispherical shape pits and volcanic shape tips, etching gas and high energy density SF_6_/O_2_ plasma should not only react with the silicon in the vertical direction but also in the horizontal direction. In previous work, the etching rate ratios in the vertical direction and horizontal direction were measured by considering the different pattern sizes of the masks [70,71]. Therefore, the sizes of the masks were designed according to the results of the etching rate ratio. In fact, the No. 1 to No. 5 tip samples and the No. 1 to No. 5 pit samples were designed in one chrome photomask and, thus, ten samples (No. 1 to No. 5 tip samples and No. 1 to No. 5 pit samples) were patterned in one silicon wafer at one time. That is to say, ten samples were present in one wafer with different sizes and they can be recognized by the number order that is near them.

Different depths of first-class structures also influenced the absorption of black silicon since deeper trenches may indicate higher absorption efficiencies. Therefore, four kinds of depth were designed, as shown in Table 2. Because the masks were different, the etching rate of the pits was different in the four different depths. Therefore, the design depth would be different in the same row. #1–#4 denote the four numbers of the different depths and 1–5 denote the five numbers of the different masks. In order to distinguish the size parameters shown in Table 1, the depth parameters are named No. #1 to No. #8. In fact, each group among No. #1 to No. #8 denote one etch process at one time. However, due to the different sizes of masks shown in Table 1, the No. 1 to No. 5 tip and pits samples might show different results of depth, which were studied in previous research [70,71]. Therefore, Table 2 indicates the design number of the depths of each sample in Table 1.

**Figure 4:**
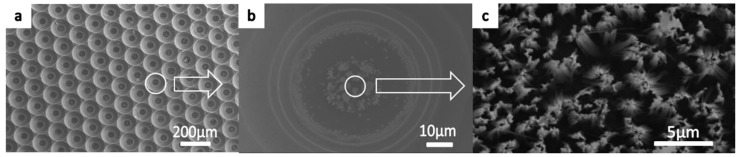
The nanostructures on the micro pits obtained using the wet etching method: (**a**) The top view of the low-magnification micrograph, (**b**) silver on the bottom surface of the pits, and (**c**) the high-magnification SEM image of nanostructures on the bottom surface of the pits.

During the process, some problems occurred in the bottom of the pits. Figure 4a illustrates the nanostructures on the micro pits obtained using the wet etching method. In contrast to the tips, the nanostructures were hardly observed on the bottom of the pits, although some silver was deposited on the bottom, as shown in Figure 4b. Figure 4c shows a high-magnification SEM image, indicating the nanostructures on the bottom surface of the pits. During fabrication, many bubbles were observed and remained in the pit area rather than diffusing away. It is possible that nanostructures were not formed easily on the bottom of the pits because the bubbles hinder or slow the chemical reaction since the etching end product is SiF_4_, a type of gas that requires air for diffusion.

**Figure 5:**
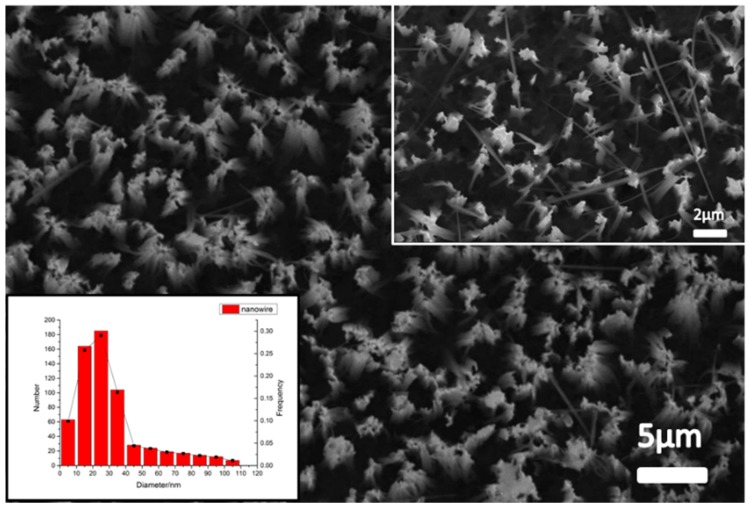
The nanostructures SEM image and frequency, and the number distribution histogram of the nanostructures.

The bottom-left inset of Figure 5 indicates that the diameter of the nanostructures was variable and the SEM images of the nanostructures are also shown in the same figure. By using a statistical image processing method, the frequency and the number distribution are plotted in the distribution histogram. The nanostructures with diameters between 20 to 30 nm were in the majority and diameters increasing from 30 nm had an ever-decreasing frequency. Nanostructures with a diameter between 10 to 40 nm accounted for 81% of all diameters.

**Figure 6:**
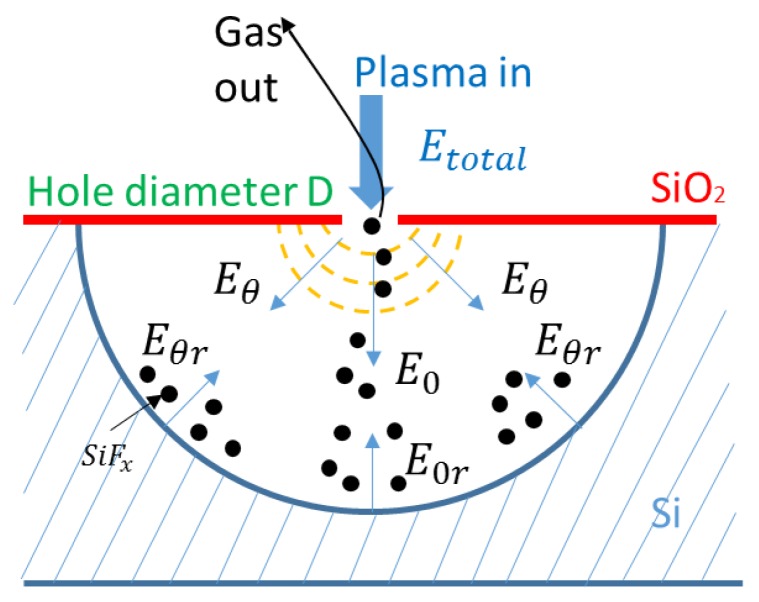
The schematic of the reaction in a microhole.

What is more, it can be inferred that the etching reaction was extremely slow or even stopped in very small mask holes: the No. 1 sample of pits. This may be interpreted by the principle shown in Figure 6. During the etching process, SiF_x_ will be produced and pumped out from the small hole. Since the diameter of the small hole (*D* in the figure) reaches the scale of electromagnetic wavelength, plasma may occur in a phenomenon that is similar to diffraction, as the dotted yellow line shown in Figure 6.

Both plasma and the reaction gas flow through the small hole called the throat of the channel. After diffraction and collision with the small hole, the total energy would be consumed to an extended value. Therefore, the total energy can be calculated by Equation (1).

(1) $$E_{total}=\int_{-\frac{\pi }{2}}^{\frac{\pi }{2}}\alpha E_{\theta }d\theta$$

where $E_{total}$ denotes the total energy of plasma, $E_{\theta }$ denotes the direction energy component in the θ angle, and α denotes the coefficient of energy loss after diffraction and collision.

Because of the throat effect, the reaction gas may not be pumped out immediately and it may stay in the trench, which results in more collisions and, thus, causes more energy loss. This may be why the etching rate is so slow with a small hole mask. Therefore, the eventual plasma with enough energy to reach the silicon surface can be described by Equation (2).

(2) $$E_{reaction}=\int_{-\frac{\pi }{2}}^{\frac{\pi }{2}}\alpha E_{\theta }-E_{\theta r}d\theta$$

where $E_{reaction}$ denotes the energy of the plasma that could react, $E_{\theta }$ denotes the direction energy component in the θ angle, $E_{\theta r}$ denotes the direction component of the loss energy in the θ angle, and α denotes the coefficient of energy loss after diffraction and collision.

Therefore, the throat effect not only influences the etching rate ratios in the vertical direction and horizontal direction but may also stop the etching process to a certain depth. As shown in Table 3, the depth and width of the trench remained at 18.2 μm and 18.5 μm, respectively.

## 3. Experimental

The actual etching data are shown in Table 3. Apart from the depth data, there are width data from the pits part. The pits’ trenches were difficult to control since they have very small mask holes, ranging from 4.2 to 50.4 μm. During the etching process, reactive plasma would be grabbed from small trenches to big ones due to the faster reaction and mass transfer in bigger reaction spaces. In addition, the etching rate ratios in the vertical direction and horizontal direction would decrease with the diameter reduction of the mask hole, as shown in Table 3.

**Figure 7:**
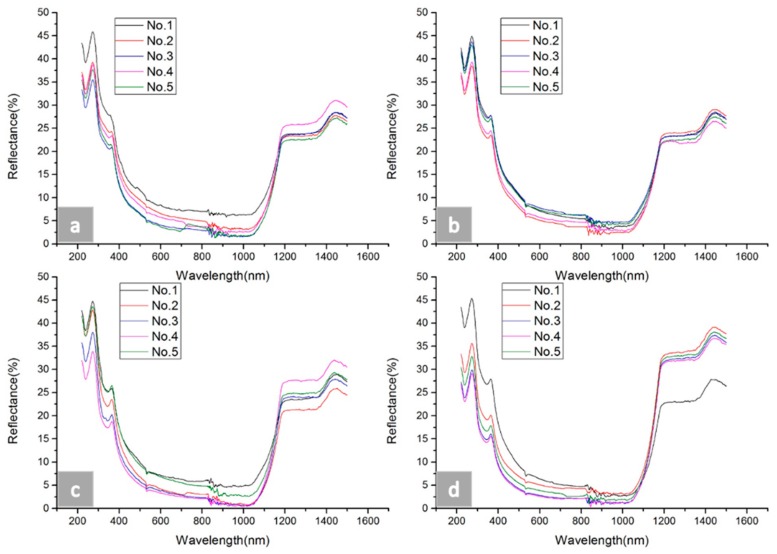
The hemispherical reflectance spectra of black silicon micro-nano hybrid structures obtained for different tips size structures. (**a**) The reflectance results of the five samples with #1 depth size, (**b**) the reflectance results of the five samples with #2 depth size, (**c**) the reflectance results of the five samples with #3 depth size, and (**d**) the reflectance results of the five samples with #4 depth size.

In this paper, nanostructures were fabricated on microstructures with different profiles: tip and pit. The microstructures were dry etched in SF_6_/O_2_ plasma using an ICP (inductively coupled plasma) from the SPTS Company (Newport, UK). Black silicon nanostructures were fabricated by the metal-assisted wet etching method, where silver is used as a catalyst. After the black silicon nanostructures were grown, the samples were observed by a photometer for reflectivity tests. As shown in Figure 7, four figures are presented, and the reflectivity values of the samples are illustrated. These samples were fabricated with #1–#4 sizes as shown in Table 3. No. 1–No. 5 denote the results of reflectance with different distances in the same depth, as shown in Table 3. In the four figures, the reflectance rapidly dropped from wavelengths of 220 nm to 600 nm, maintained a relatively low value from wavelengths of 600 nm to 1050 nm, and reached the lowest value at a wavelength of 1000 nm. However, the reflectance sharply jumped at a wavelength of 1050 nm. A fluctuation could be seen near the wavelengths of 880 nm, which may have been caused by instrument vibration.

**Figure 8:**
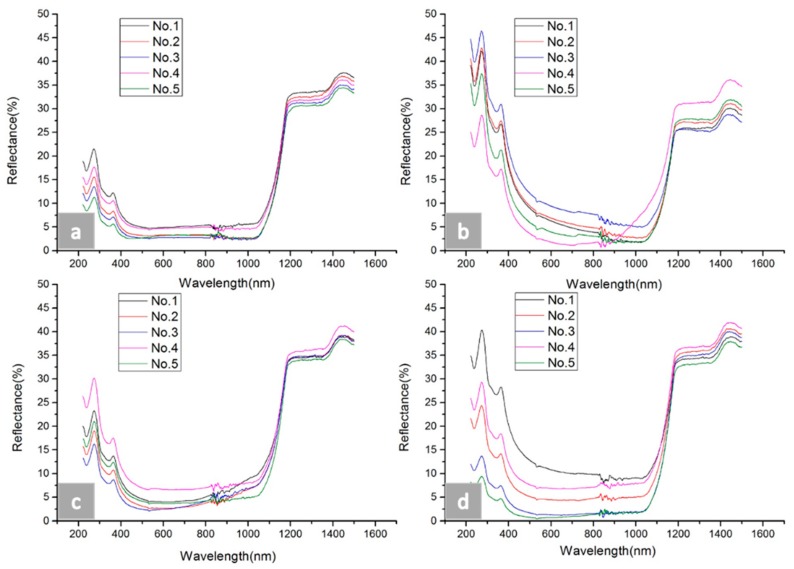
The hemispherical reflectance spectra of the black silicon micro-nano hybrid structures obtained for the different pits size structures. (**a**) The reflectance results of the five samples with #5 depth size, (**b**) the reflectance results of the five samples with #6 depth size, (**c**) the reflectance results of the five samples with #7 depth size, and (**d**) the reflectance results of the five samples with #8 depth size.

As shown in Figure 8, four graphs are presented, and the reflectivity values of the samples are illustrated. These samples were fabricated with #5–#8 sizes, as shown in Table 3. No. 1–No. 5 denote the results of reflectance with different distances in the same depth, as shown in Table 3. In the four graphs, the reflectance dropped from wavelengths of 220 nm to 400 nm, maintained a relatively low value from wavelengths of 400 nm to 1050 nm, and sharply jumped at a wavelength of 1050 nm. However, the lowest value of reflectance could not be distinguished easily since the values of low reflectance remained equable. A fluctuation could also be seen near a wavelength of 880 nm, which may have been caused by instrument vibration.

**Figure 9:**
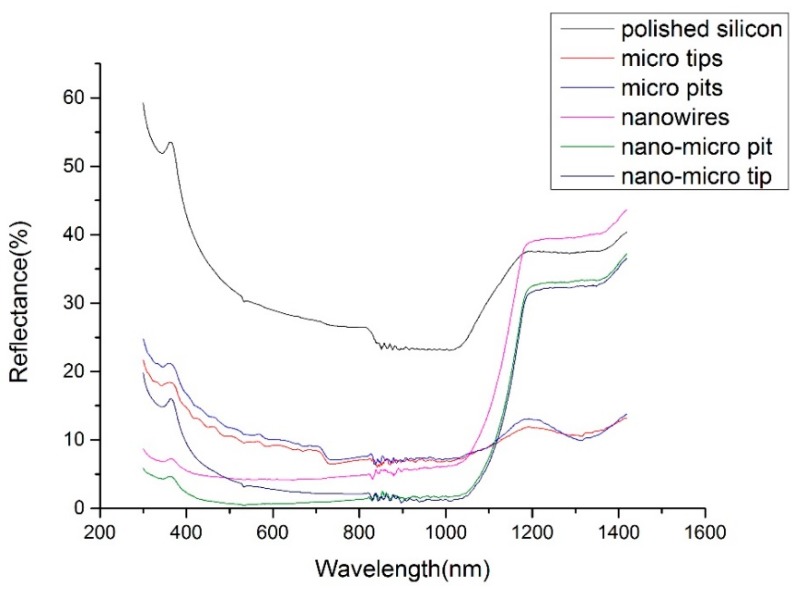
The reflectance spectrum results of polished silicon, micro tips, micro pits, nanowires nano-micro pit, and nano-micro tip structures.

As shown in Figure 9, six reflectance spectrums were indicated and compared. The black line indicates that the polished silicon without micro and nanostructures showed the highest reflectance. The surfaces with microstructures (micro tips or pits) had lower reflectance values, though higher than those with single nanowires. With hybrid structures, the nano-micro pits and tips showed extremely low reflectance values. In addition, the former one showed the lowest reflectance.

## 4. Results and Discussion

**Figure 10:**
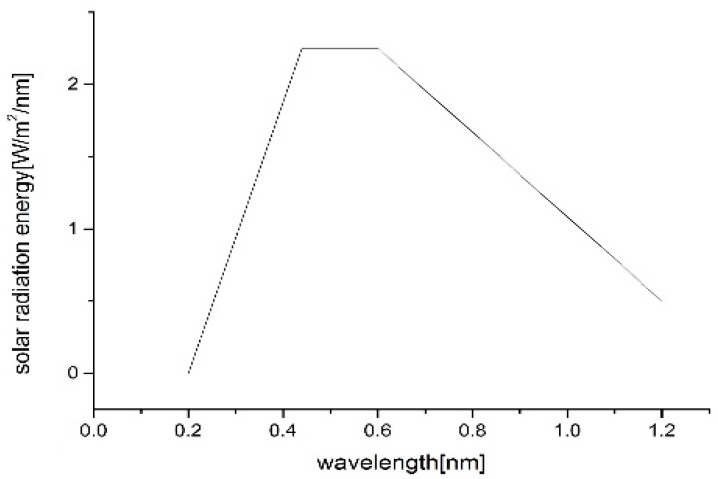
The approximate curve of actual solar radiation energy on the Earth’s surface from 200 nm to 1200 nm.

Based on the actual solar radiation energy at the Earth’s surface [72], an approximate curve is illustrated in Figure 10, which consists of three line segments, to simplify the absorption efficiency of the six micro-nano hybrid black silicon samples and single microstructures samples. The equation of the absorption efficiency is given below:

(3) $$A=\int_{300}^{1100}\left(1-R\left(\lambda \right)\right)\times E\left(\lambda \right)\;d\lambda$$

where *A* is the absorption efficiency, *R*(*λ*) is the reflectance of black silicon at wavelength *λ*, and E(λ) is the actual solar radiation energy at the Earth’s surface for black silicon at wavelength *λ*. For the range of integration (300−1100 nm), the minimum value was limited by the lowest wavelength value of the photometer, and the maximum value was limited by the forbidden gap of silicon.

**Figure 11:**
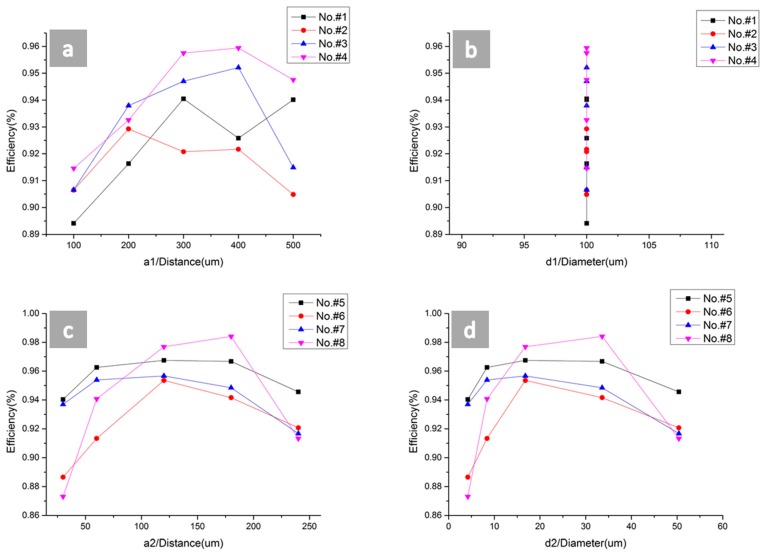
The absorption efficiency of the micro-nano hybrid structures obtained for different tip and pit sizes. (**a**) The relational graph between the efficiency and diameter of the tips (first designed trench size) and (**b**) the relational graph between the efficiency and depth of the tips (first designed trench size). (**c**) The relational graph between the efficiency and diameter of the pits (first designed trench size) and (**d**) the relational graph between the efficiency and depth of the pits (first designed trench size).

As shown in Figure 11a, the sample with a distance of 400 μm and a depth of 48 μm exhibited the highest absorption efficiency among the tip samples, and the sample with a distance of 100 μm and a depth of 24 μm exhibited the lowest absorption efficiency among the tip samples. The efficiency of tip samples increased firstly and then decreased as the distance changes. This was because too short of a distance would cause the nanostructures to hardly grow on the plates between the two tips and too long of a distance would decrease the number of tips, which would reduce the absorption efficiency. In general, the efficiency of the tip samples with a high depth was higher than those with a low depth since a deeper trench denoted a darker screen. The highest efficiency of the samples exceeded the lowest efficiency of the samples by almost 6.5%. As shown in Figure 11b, it seems like a line. However, it has four depths (No. #1 to No. #4) lines and 20 points because *d_1_* remains the same value, one that is different from Figure 11d. However, the maximum and minimum values of different sizes can be found in Figure 11b. The range was more than 6%.

As shown in Figure 11c,d, the diameter and depth values are the first trench size. The sample with a diameter of 117.1 μm and a depth of 72.6 μm exhibited the highest absorption efficiency among the pit samples and the sample with a diameter of 18.5 μm and a depth of 18.2 μm exhibited the lowest absorption efficiency among the pit samples. The efficiency of the tip samples increased firstly and then decreased as the diameter or depth changes. This was because too small of a diameter would cause nanostructures to hardly grow on the bottom of the pits and too large of a diameter would decrease the number of pits, which would reduce the absorption efficiency. The highest efficiency of the samples exceeded the lowest efficiency of the samples by almost 11%.

## 5. Conclusions

This paper formed a novel secondary nanostructured black silicon, which used the dry-wet hybrid fabrication method to achieve secondary nanostructures. This paper fabricated different sizes of secondary nanostructured black silicon and compared their absorptions with each other. The best absorption performance of black silicon came from the combination of micro first-class structures and nano secondary structures. A 0.5% reflectance and 98% absorption efficiency of the pit sample were achieved with a diameter of 117.1 μm and a depth of 72.6 μm. In addition, the variation tendency of the absorption efficiency was not solely monotone increasing or monotone decreasing, but firstly increasing and then decreasing. By using a statistical image processing method, nanostructures with diameters between 20 to 30 nm were in the majority and nanostructures with diameters between 10 to 40 nm accounted for 81% of all diameters. The effect of etching stop was interpreted and analyzed.

## Figures and Tables

**Table 1:** The size details of the tips mask and pits mask.

Number	Tips		Pits	
	*a*_1_ (μm)	*d*_1_ (μm)	*a*_2_ (μm)	*d*_2_ (μm)
1	100	100	30	4.2
2	200	100	60	8.4
3	300	100	120	16.8
4	400	100	180	33.6
5	500	100	240	50.4

**Table 2:** The design table of four kinds of depth of tips and pits.

**Tip Sample Number**	**#1 (Depth) μm**	**#2 (Depth) μm**	**#3 (Depth) μm**	**#4 (Depth) μm**
No. 1	24	32	40	48
No. 2	24	32	40	48
No. 3	24	32	40	48
No. 4	24	32	40	48
No. 5	24	32	40	48
**Pit Sample Number**	**#5 (Depth) μm**	**#6 (Depth) μm**	**#7 (Depth) μm**	**#8 (Depth) μm**
No. 1	20	21	22	23
No. 2	30	32	34	36
No. 3	40	43	46	49
No. 4	50	56	62	68
No. 5	60	68	76	84

**Table 3:** The actual etching data table of the four kinds of depth and width of the tips and pits.

**Tips**	**#1 (Depth) μm**	**#2 (Depth) μm**	**#3 (Depth) μm**	**#4 (Depth) μm**
1	24.2	32.2	40.1	47.9
2	24.3	32.3	40.2	47.6
3	24.1	32.2	40.2	47.8
4	24.4	32.3	40.1	47.6
5	24.2	32.1	40.3	47.7
**Pits**	**#5 (width/depth) μm**	**#6 (width/depth) μm**	**#7 (width/depth) μm**	**#8 (width/depth) μm**
1	18.2/17.9	18.3/18.1	18.5/18.2	18.5/18.2
2	22.2/23.0	27.8/31.1	26.8/33.0	32.8/33.6
3	56.9/35.2	46.6/42.2	51.7/44.8	69.3/46.7
4	87.4/52.0	96.3/61.3	107.9/63.7	117.1/72.6
5	109.7/55	127.3/69.3	140.9/80.0	148.9/83.7

## References

[1] Jansen H., Boer M.D., Burger J., Legtenberg R., Elwenspoek M. (1995). The black silicon method II: The effect of mask material and loading on the reactive ion etching of deep silicon trenches. Microelectron. Eng..

[2] Ruby D.S., Zaidi S.H., Narayanan S., Bathey B. (2005). RIE-texturing of industrial multicrystalline silicon solar cells. J. Sol. Energy Eng..

[3] Bhadra C.M., Werner M., Baulin V.A., Truong V.K., Al Kobaisi M., Nguyen S.H., Balcytis A., Juodkazis S., Wang J.Y., Mainwaring D.E. (2018). Subtle Variations in Surface Properties of Black Silicon Surfaces Influence the Degree of Bactericidal Efficiency. Nano-Micro Lett..

[4] Linklater D.P., Nguyen H.K.D., Bhadra C.M., Juodkazis S., Ivanova E.P. (2017). Corrigendum: Influence of nanoscale topology on the bactericidal efficiency of black silicon surfaces. Nanotechnology.

[5] Steglich M., Käsebier T., Zilk M., Pertsch T., Kley E.B., Tünnermann A. (2014). The structural and optical properties of black silicon by inductively coupled plasma reactive ion etching. J. Appl. Phys..

[6] Dussart R., Mellhaoui X., Tillocher T., Lefaucheux P., Volatier M., Socquet-Clerc C., Brault P., Ranson P. (2005). Silicon columnar microstructures. J. Phys. D Appl. Phys..

[7] Pezoldt J., Kups T., Stubenrauch M., Fischer M. (2011). Black luminescent silicon. Phys. Status Solidi.

[8] Yuan H.C., Yost V.E., Page M.R., Stradins P., Meier D.L., Branz H.M. (2009). Efficient black silicon solar cell with a density-graded nanoporous surface: Optical properties, performance limitations, and design rules. Appl. Phys. Lett..

[9] Toor F., Branz H.M., Page M.R., Jones K.M., Yuan H.C. (2011). Multi-scale surface texture to improve blue response of nanoporous black silicon solar cells. Appl. Phys. Lett..

[10] Nishioka K., Sueto T., Saito N. (2009). Formation of antireflection nanostructure for silicon solar cells using catalysis of single nano-sized silver particle. Appl. Surf. Sci..

[11] Koynov S., Brandt M.S., Stutzmann M. (2007). Black multi-crystalline silicon solar cells. Phys. Status Solidi (RRL)—Rapid Res. Lett..

[12] Branz H.M., Yost V.E., Ward S., Jones K.M. (2009). Nanostructured black silicon and the optical reflectance of graded-density surfaces. Appl. Phys. Lett..

[13] Zhou Z.Q., Hu F., Zhou W.J., Chen H.Y., Ma L., Zhang C., Lu M. (2017). An Investigation on a Crystalline-Silicon Solar Cell with Black Silicon Layer at the Rear. Nanoscale Res. Lett..

[14] Mitsai E., Kuchmizhak A., Pustovalov E., Sergeev A., Mironenko A., Bratskaya S., Linklater D.P., Balčytis A., Ivanova E., Juodkazis S. (2018). Chemically non-perturbing SERS detection of catalytic reaction with black silicon. Nanoscale.

[15] Li J.Y., Hung C.H., Chen C.Y. (2017). Hybrid black silicon solar cells textured with the interplay of copper-induced galvanic displacement. Sci. Rep..

[16] Halima A.F., Zhang X., Macfarlane D.R. (2017). Metal-Free Black Silicon for Solar-powered Hydrogen Generation. Electrochim. Acta.

[17] Rahman T., Boden S.A. (2017). Optical Modeling of Black Silicon for Solar Cells Using Effective Index Techniques. IEEE J. Photovolt..

[18] Jia X., Zhou C., Wang W. (2017). Optimization of the Surface Structure on Black Silicon for Surface Passivation. Nanoscale Res. Lett..

[19] Su G., Jia R., Dai X., Tao K., Sun H., Jin Z., Liu X. (2018). The Influence of Black Silicon Morphology Modification by Acid Etching to the Properties of Diamond Wire Sawn Multicrystalline Silicon Solar Cells. IEEE J. Photovolt..

[20] Hsu C.H., Wu J.R., Lu Y.T., Flood D.J., Barron A.R., Chen L.C. (2014). Fabrication and characteristics of black silicon for solar cell applications: An overview. Mater. Sci. Semicond. Process..

[21] Liu X., Coxon P.R., Peters M., Hoex B., Cole J.M., Fray D.J. (2014). Black silicon: Fabrication methods, properties and solar energy applications. Energy Environ. Sci..

[22] Hirsch J., Gaudig M., Bernhard N., Lausch D. (2016). Optoelectronic properties of black-silicon generated through inductively coupled plasma (icp) processing for crystalline silicon solar cells. Appl. Surf. Sci..

[23] Kotsifaki D.G., Kandyla M., Lagoudakis P.G. (2016). Plasmon enhanced optical tweezers with gold-coated black silicon. Sci. Rep..

[24] Pasanen T., Vähänissi V., Theut N., Savin H. (2017). Surface passivation of black silicon phosphorus emitters with atomic layer deposited sio2/al2o3 stacks. Energy Procedia.

[25] Yang L.X., Chao Y.M., Jia L., Li C.B. (2016). Wettability and boiling heat transfer study of black silicon surface produced using the plasma immersion ion implantation method. Appl. Therm. Eng..

[26] Cai W., Xiong H., Su X., Zhou H., Shen M., Fang L. (2017). Enhanced photoelectrochemical properties of copper-assisted catalyzed etching black silicon by electrodepositing cobalt. Appl. Phys. Lett..

[27] An E.L., Lim C.Y., Lam Y.C., Taboryski R. (2018). *Electroosmotic* flow in microchannel with black silicon nanostructures. Micromachines.

[28] Lv J., Zhang T., Zhang P., Zhao Y., Li S. (2018). Review Application of Nanostructured Black Silicon. Nanoscale Res. Lett..

[29] Crouch C.H., Carey J.E., Shen M., Mazur E., Genin F.Y. (2004). Infrared absorption by sulfurdoped silicon formed by femtosecond laser irradiation. Appl. Phys. A.

[30] Carey J.E., Crouch C.H., Shen M., Mazur E. (2005). Visible and near-infrared responsivity of femtosecond-laser microstructured silicon photodiodes. Opt. Lett..

[31] Torres R., Itina T.E., Vervisch V., Halbwax M., Derrien T., Sarnet T., Sentis M., Ferreira J., Torregrosa F., Roux L. (2010). Study on laser-induced periodic structures and photovoltaic application. AIP Conf. Proc..

[32] Aoife M., Moloney L.W., Mathewson A., Healy G., Carlton Jackson J. (2006). Study of the properties of new SPM detectors. Semiconductor photodetectors III. Int. Soc. Opt. Photonics.

[33] Zhang S.K., Ahmar H., Chen B., Wang W., Alfano R. (2011). Photocurrent spectrum measurements of doped black silicon. Proc. SPIE.

[34] Carey J.E., Mazur E. High sensitivity silicon-based VIS/NIR photodetectors. Proceedings of the Conference on Lasers and Electro-Optics.

[35] Huang Z., Carey J.E., Liu M., Guo X., Mazur E., Campbell J.C. (2006). Microstructured silicon photodetector. Appl. Phys. Lett..

[36] Zhao Y., Lai J., Zhou H., Yi X. (2004). Fabrication of anisotropic structures with large aspect ratio and minimal roughness by using black silicon method. Proc. SPIE.

[37] Pralle M.U., Carey J.E., Homayoon H., Alie S., Sickler J., Li X., Jiang J., Miller D., Palsule C., McKee J. (2010). Black silicon enhanced photodetectors: A path to IR CMOS. Proc. SPIE.

[38] Halbwax M., Sarnet T., Delaporte P., Sentis M., Etienne H., Torregrosa F., Vervisch V., Perichaud I., Martinuzzi S. (2008). Micro and nano-structuration of silicon by femtosecond laser: Application to silicon photovoltaic cells fabrication. Thin Solid Films.

[39] Zhang T., Ahmad W., Liu B., Xuan Y., Ying X., Liu Z., Li Y., Chen Z., Li S. (2017). Broadband infrared response of sulfur hyperdoped silicon under femtosecond laser irradiation. Mater. Lett..

[40] Zhang T., Liu B., Ahmad W., Xuan Y., Ying X., Liu Z., Chen Z., Li S. (2017). Optical and electronic properties of femtosecond laser-induced sulfur-hyperdoped silicon N+/P photodiodes. Nanoscale Res. Lett..

[41] Yuan H.C., Yost V.E., Page M.R., Roybal L., To B., Stradins P., Meier D.L., Branz H.M. Efficient black silicon solar cells with nanoporous anti-reflection made in a single-step liquid etch. Proceedings of the 34th IEEE Photovoltaic Specialists Conference (PVSC).

[42] Srivastava S.K., Kumar D., Singh P.K., Kumar V. Silicon nanowire arrays based “black silicon” solar cells. Proceedings of the 34th IEEE Photovoltaic Specialists Conference (PVSC).

[43] Sarnet T., Halbwax M., Torres R., Delaporte P., Sentis M., Martinuzzi S., Vervisch V., Bastide S. (2008). Femtosecond laser for black silicon and photovoltaic cells. Proc. SPIE.

[44] Hu L., Chen G. (2007). Analysis of optical absorption in silicon nanowire arrays for photovoltaic applications. Nano Lett..

[45] Wang W., Wu S., Reinhardt K., Lu Y., Chen S. (2010). Broadband light absorption enhancement in thin-film silicon solar cells. Nano Lett..

[46] Garnett E., Yang P. (2010). Light trapping in silicon nanowire solar cells. Nano Lett..

[47] Yan Y., Yuan H.C., Yost V.E., Jones K., Al-Jassim M., Branz H.M. Microstructure and surface chemistry of nanoporous “black silicon” for photovoltaics. Proceedings of the 2010 35th IEEE Photovoltaic Specialists Conference (PVSC).

[48] Flory F., Escoubas L., Berginc G. (2011). Optical properties of nanostructured materials: A review. J. Nanophotonics.

[49] Scotti G., Kanninen P., Maakinen M., Kallio T., Franssila S. (2010). Silicon nanograss as micro fuel cell gas diffusion layer. Micro Nano Lett..

[50] Crouch C.H., Mazur E., Carey J.E. (2003). Femtosecond-Laser-Assisted Microstructuring of Silicon Surfaces. Opt. Photonics News.

[51] Carey J.E., Crouch C.H., Younkin R., Mazur E., Sheehy M., Friend C. Fabrication of micrometer-sized conical field emitters using femtosecond laser-assisted etching of silicon. Proceedings of the 14th Vacuum Microelectronics Conference.

[52] Hoyer P., Theuer M., Beigang R., Kley E.-B. (2008). Terahertz emission from black silicon. Appl. Phys. Lett..

[53] Li X., Bohn P.W. (2000). Metal-assisted chemical etching in HF/H_2_O_2_ produces porous silicon. Appl. Phys. Lett..

[54] Kurumurthy G., Alee K.S., Rao D.N. (2009). Photoluminescence studies of Si/SiO2 nanoparticles synthesized with different laser irradiation wavelengths of nanosecond pulse duration. Opt. Commun..

[55] Zorba V., Persano L., Pisignano D., Athanassiou A., Stratakis E., Cingolani R., Tzanetakis P., Fotakis C. (2006). Making silicon hydrophobic: Wettability control by two-lengthscale simultaneous patterning with femtosecond laser irradiation. Nanotechnology.

[56] Cao L., Jones A.K., Sikka V.K., Wu J., Gao D. (2009). Anti-icing superhydrophobic coatings. Langmuir.

[57] Zhu J., Hsu C.M., Yu Z., Fan S., Cui Y. (2010). Nanodome solar cells with efficient light management and self-cleaning. Nano Lett..

[58] Barthlott W., Neinhuis C. (1997). Purity of the sacred lotus, or escape from contamination in biological surfaces. Planta.

[59] Li C.H., Wang X.P., Zhao J.H., Zhang D.Z., Yu X.Y., Li X.B., Feng J., Chen Q.D., Ruan S.P., Sun H.B. (2018). Black silicon IR photodiode supersaturated with nitrogen by femtosecond laser irradiation. IEEE Sens. J..

[60] Zhan X., Xu H., Li C., Zang H., Liu C., Zhao J., Sun H. (2017). Remote and rapid micromachining of broadband low-reflectivity black silicon surfaces by femtosecond laser filaments. Opt. Lett..

[61] Li C.H., Zhao J.H., Chen Q.D., Feng J., Sun H.B. (2018). Sub-bandgap photo-response of non-doped black-silicon fabricated by nanosecond laser irradiation. Opt. Lett..

[62] Kim B.S., Shin S., Shin S.J., Kim K.M., Cho H.H. (2011). Micro-nano hybrid structures with manipulated wettability using a two-step silicon etching on a large area. Nanoscale Res. Lett..

[63] Venkatesan R., Arivalagan M.K., Venkatachalapathy V., Pearce J.M., Mayandi J. (2018). Effects of silver catalyst concentration in metal assisted chemical etching of silicon. Mater. Lett..

[64] Shen Z., Liu B., Xia Y., Liu J., Liu J., Zhong S., Li C. (2013). Black silicon on emitter diminishes the lateral electric field and enhances the blue response of a solar cell by optimizing depletion region uniformity. Scr. Mater..

[65] Park H., Shin D., Kang G., Baek S., Kim K., Padilla W.J. (2011). Broadband optical antireflection enhancement by integrating antireflective nanoislands with silicon nanoconical-frustum arrays. Adv. Mater..

[66] Zhou Z., Sakr E., Sun Y., Bermel P. (2016). Solar thermophotovoltaics: Reshaping the solar spectrum. Nanophotonics.

[67] Lee K., Hwang I., Kim N., Choi D., Um H.D., Kim S., Seo K. (2016). 17.6%-Efficient radial junction solar cells using silicon nano/micro hybrid structures. Nanoscale.

[68] Park K.T., Guo Z., Um H.D., Jung J.Y., Yang J.M., Lim S.K., Kim Y.S., Lee J.H. (2011). Optical properties of Si microwires combined with nanoneedles for flexible thin film photovoltaics. Opt. Express.

[69] Lee I.J., Paik U., Park J.G. (2013). Solar cell implemented with silicon nanowires on pyramid-texture silicon surface. Sol. Energy.

[70] Xu T., Tao Z., Li H., Tan X., Li H. (2017). Effects of deep reactive ion etching parameters on etching rate and surface morphology in extremely deep silicon etch process with high aspect ratio. Adv. Mech. Eng..

[71] Tan X., Tao Z., Yu M., Wu H., Li H. (2018). Anti-reflectance investigation of a micro-nano hybrid structure fabricated by dry/wet etching methods. Sci. Rep..

[72] Xu Y., Liu H., Xu J., Liu H. (2000). An Introduction to Atmospheric Science.

